# Time of admission and mortality after hip fracture: a detailed look at the weekend effect in a nationwide study of 55,211 hip fracture patients in Norway

**DOI:** 10.1080/17453674.2018.1533769

**Published:** 2018-11-06

**Authors:** Andreas Asheim, Sara Marie Nilsen, Marlen Toch-Marquardt, Kjartan Sarheim Anthun, Lars Gunnar Johnsen, Johan Håkon Bjårngaard

**Affiliations:** 1 Center for Health Care Improvement, St Olav’s HospitalS, Trondheim, Norway;;; 2 Norwegian University of Science and Technology, Department of Mathematical Sciences, Trondheim, Norway;;; 3 Norwegian University of Science and Technology, Department of Public Health and Nursing, Trondheim, Norway;;; 4 Department of Health Research, SINTEF Digital, Trondheim, Norway;; 5 Department of Orthopaedic Surgery, St Olav’s Hospital, Trondheim, Norway;;; 6 Norwegian University of Science and Technology, Department of Neuromedicine, Trondheim, Norway

## Abstract

Background and purpose — There are numerous studies on the weekend effect for hip fracture patients, with conflicting results. We analyzed time of admission and discharge, and the association with mortality and length of hospital stay in more detail.

Patients and methods — We used data from 61,211 surgically treated hip fractures in 55,211 patients, admitted to Norwegian hospitals 2008–2014. All patients were aged 50 years or older. Data were analyzed with Cox and Poisson regression.

Results — Mortality within 30 days did not differ substantially by day of admission, although admissions on Sundays and holidays had a slightly increased mortality. The hazard ratios were 1.1 (95% confidence interval [CI] 0.97–1.2) for Sundays, and 1.2 (CI 0.98–1.4) for holidays, relative to Mondays. For patients admitted between 6:00 am and 7:00 am the hazard ratio was 1.4 (CI 1.1–1.8) relative to patients admitted between 2:00 pm and 3:00 pm. Discharges during weekends and holidays were associated with a substantial higher mortality than weekday discharges. Patients admitted from Friday to Sunday generally stayed in hospital for a shorter time than patients admitted during other days.

Interpretation — Our results indicate that the discussion on weekday versus weekend admission effects might have distracted attention from other important factors, such as time of day of admission, and day of discharge from hospital treatment.

Despite improvements in health care organization and medical technology, 1 out of 10 hip fracture patients die within 30 days after admission (Giannoulis et al. 2016, Søgaard et al. 2016, Johansen et al. 2017).

With hip fractures being acute, an event occurring out-of-hours will pose a challenge to the health care system. While increased mortality for patients admitted at weekends relative to weekdays has been reported by some studies (Thomas et al. 2014, Kristiansen et al. 2016), others have not found this (Foss and Kehlet 2006, Daugaard et al. 2012, Boylan et al. 2015, Nandra et al. 2017, Nijland et al. 2017, Sayers et al. 2017). These studies had limited information on the volume of admissions by day of the week and time of day, and most included few cases.

Most of these studies are limited to aggregation of information on timing into binary indicators of weekdays versus weekends, and out-of-hours versus working hours (Sayers et al. 2017). However, these are just two of several patterns of time variation, which as well are likely to vary by clinical pathways and health care settings (Bray et al. 2016). A recent Danish study (Sayers et al. 2017) on hip fractures, for example, reported a 17% higher mortality for out-of-hours discharges and 50% higher mortality for discharges on Sundays.

By using a large, nationwide cohort, we were able to describe the timing of admission and discharge, and the subsequent association with mortality and length of hospital stay.

## Patients and methods

We retrieved a nationwide cohort of 61,211 hip fractures from 55,211 unique patients, discharged from January 1, 2008 to December 31, 2014, from the Norwegian patient registry. All Norwegian hospital trusts are obliged to submit information about their activity to this registry. Each patient has a unique, anonymous identification number throughout the observation period (Hassani et al. 2015). Information on the date of death from the National Registry in Norway was provided through the Norwegian patient registry. Population-level data (e.g., population size) were obtained from Statistics Norway. Two Norwegian validation studies have shown high accuracy for hip fracture incidents (Høiberg et al. 2014, Helgeland et al. 2016), the data were also assessed as adequate for calculating 30-day mortality after hip fracture.

We identified hip fracture patients through a combination of ICD-10 codes and the Nordic Medico-Statistical Committee (NOMESCO) Classification of Surgical Procedures codes (Bay-Nielsen 2010). Admitted patients with ICD-10 codes S72.0x, S72.1x, or S72.2x as primary diagnosis and 1 or more NOMESCO codes, NFBxy (x = 0–9, y = 0–2) or NFJxy (x = 0–9, y = 0–2) during their hospitalization, were included in the study. This definition has previously shown high accuracy, excluding for example hospitalizations for rehabilitation (Høiberg et al. 2014). Patients whose hospitalization commenced with a planned admission were excluded. We also excluded re-hospitalizations within 30 days from the previous hospitalization, to avoid influence from several hospitalizations for the same hip fracture. We included patients who were registered with 1 of the relevant ICD-10 codes, but none of the procedure codes if they died during the hospitalization, to include patients who died before surgery. Due to the Norwegian coding practice, we cannot capture the non-operatively treated fractures. We selected patients aged 50 years and older. See also Figure 1 (Supplementary data) for a flow chart showing the selection criteria.

Ward and hospital transfers were considered part of the hospitalization if subsequent admissions were closer in time than 8 hours. We considered the length of hospitalization as the continuous time between admission and discharge, and not measured as discrete number of days. The construction of hospitalizations (episodes of care) was according to Hassani et al. (2015).

## Time variables

The analyses were performed using calendar variables as explanatory variables: hour, day-of-the-week/holiday, and year. Hour of admission was categorized by the number of whole hours after midnight. For hour of discharge, we used a coarser categorization, with 4 hours per category. Day-of-the-week was extended with a category named “holiday,” such that public holidays are coded as a category separate from Mondays to Sundays. The models were adjusted for age in years, without any categorization, and sex.

## Statistics

### Mortality

We used Cox regression to analyze mortality. All models used time from admission as the time scale. Patients were followed for 30 days or until time of death, whichever occurred first. We used time of admission as explanatory variable, with admission hour, day-of-the-week, month, and year. The analysis was adjusted for sex, age, and day-after-holiday. We used Mondays and hour 2:00 to 3:00 pm as reference categories, because these are categories with the largest volumes.

For the analyses of discharge day and mortality, we used the time from admission as time scale, while the risk of mortality was measured from the day of discharge to death or end of follow-up.

### Length of hospitalization

The association between time of admission and length of hospitalization was analyzed using Poisson regression with robust standard errors for patient identification, to account for dependence in the data. Admission hour, day-of-the-week, month, and year were used as explanatory variables, and the model was adjusted for sex, age, and day-after-holiday. The association between day of admission and length of hospitalization was estimated using the average marginal effect.

The analyses were performed using Stata version 14 (StataCorp, College Station, TX, USA) and RStudio version 10.5 (https://www.rstudio.com). Rates of patients in/out were computed as densities with respect to time-of-day and day-of-the-week. Detailed results from the different regression models are presented in Table 1 (Supplementary data).

## Ethics, funding, and potential conflicts of interest

The study was approved by the Regional Committee of Ethics in Medical Research (2016/2158-1). Participant consent was not required. Authors SMN and MTM were funded by the Norwegian research council (Grant number 256579). The funders had no role in study design, data collection and analysis, decision to publish, or preparation of the manuscript. The authors declare that they have no competing interests.

## Results

70% of all hip fracture incidents were among women, and the average age in our cohort was 82 years (SD 10). The 30-day case mortality after hospital admission was 8.0%. The average and median length of hospitalization were 8.6 days and 6.4 days, respectively. Table 2 (Supplementary data) shows characteristics of the portion of the cohort that died within 30 days of admission. We see a 6-year higher average age among these patients, and an overrepresentation of men. For patients who were discharged alive and died within 30 days, the length of hospitalization was around 2 days shorter compared with those who survived. In our cohort, the hip fracture incidence rate was 5.2 hip fractures per 1,000 persons per year. Table 3 (Supplementary data) includes yearly breakdowns of the incidence rate, suggesting a slight decrease from 2008 to 2014, while the 30-day case fatality remained stable. The arrival patterns were similar for all weekdays, with 41% of patients arriving between 12:00 pm and 6:00 pm (Figure 2). Saturdays and Sundays had markedly fewer patients arriving during the day, but somewhat more admissions during night-hours. Sunday had the lowest number of hip fracture admissions. For breakdowns of case fatality, sex, and age and primary diagnosis by day and hour of admission, Tables 4 and 6 (Supplementary data). Patient characteristics, sex, age, and type of fracture, vary to some extent with hour of admission but are apparently stable between days of admission.

Figure 2.Day and time of admission (left panel) and discharge (right panel).

**Figure:**
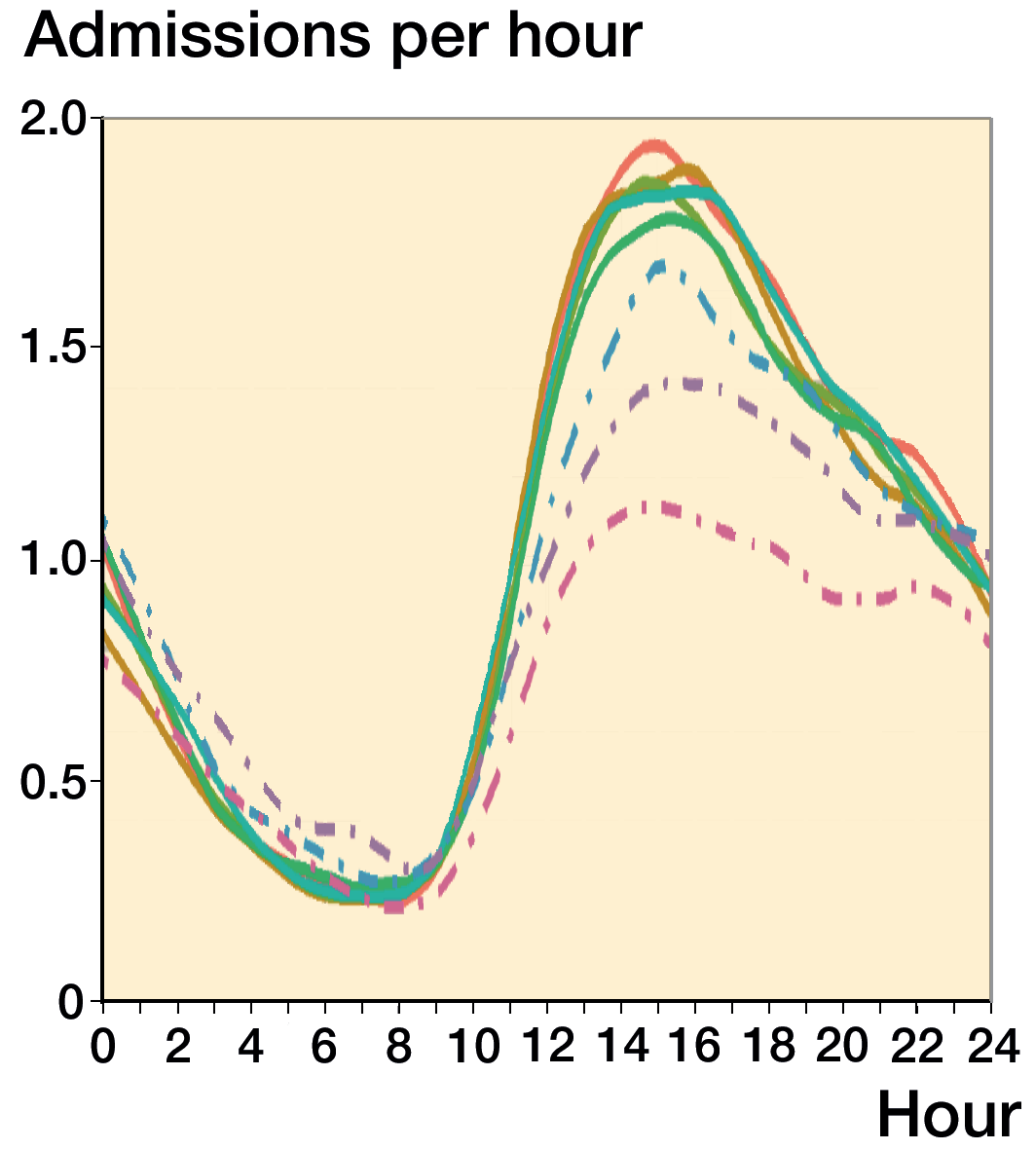

**Figure:**
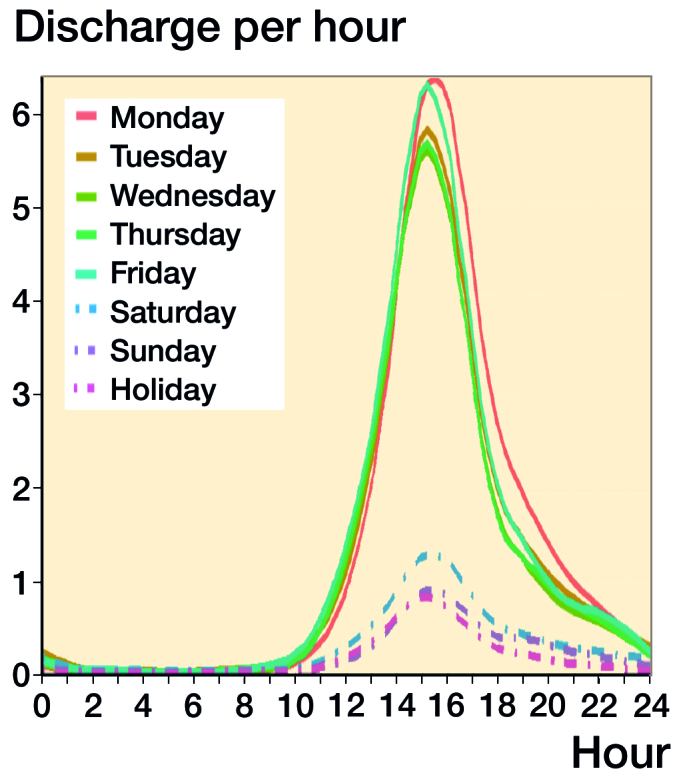

There was a tendency to higher hip fracture occurrence during the winter months December to March (Table 7, Supplementary data). The average age of patients admitted during the winter months was about 1 year lower than for the other months.

The rates of patients discharged every weekday are depicted in Figure 2. All days of the week have similar discharge patterns, where the patients were mainly discharged from 12:00 am until 6:00 pm. 74% of discharges occurred in this time span. Saturdays and Sundays had markedly fewer discharges than the other days. Holidays were almost identical to Sundays, while Mondays and Fridays had the largest volume of discharges. For information on case fatality, sex, and age by day of discharge see Table 5 (Supplementary data).

**Figure 3.**
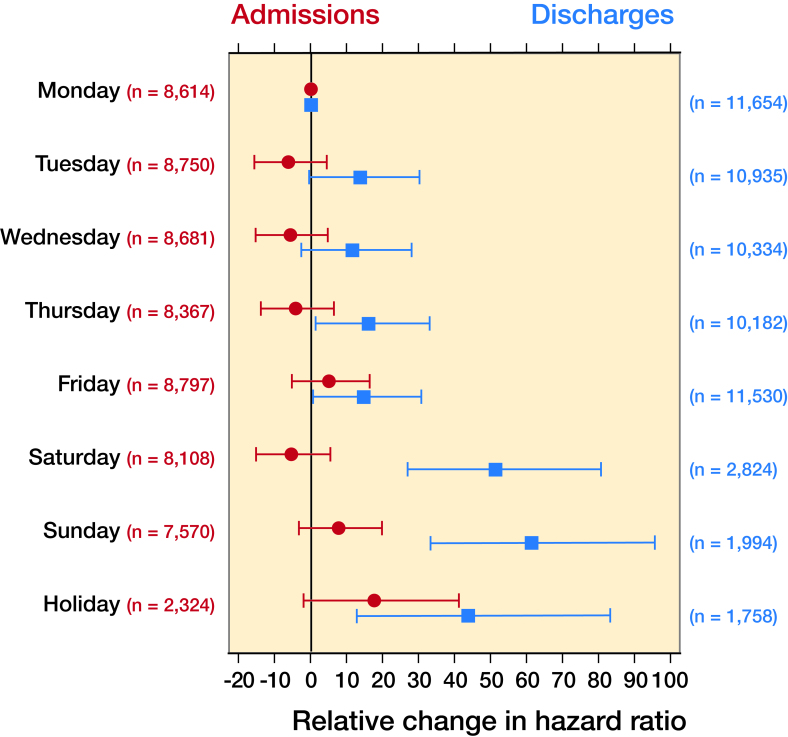
Mortality within 30 days after admission, by day of admission and discharge. Relative change in hazard ratios with 95% confidence interval is displayed. Number of admissions and discharges per category are displayed on the left and right, respectively. Adjusted for sex, age, admission hour, day-after-holiday, month, and year.

**Figure 4.**
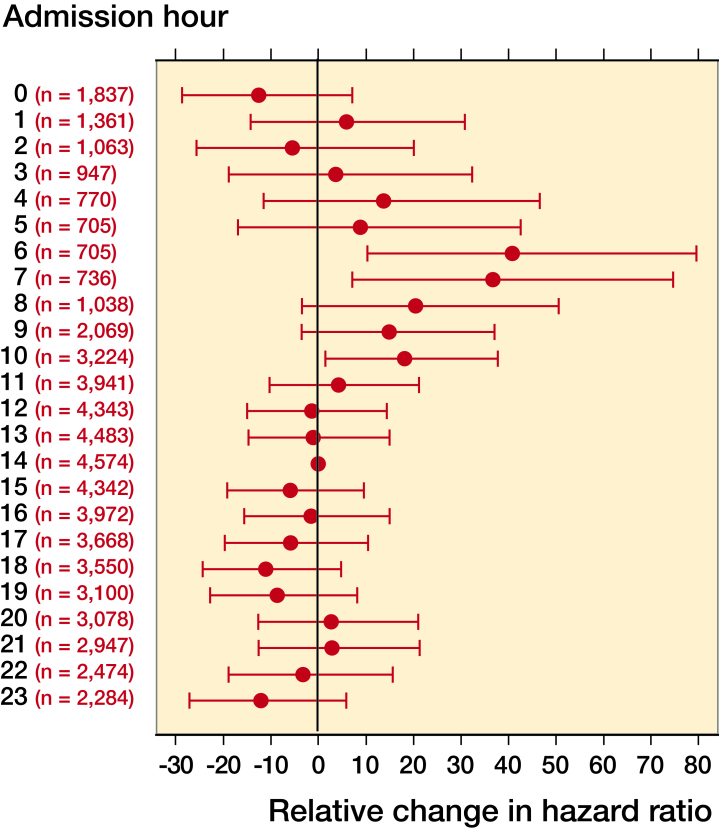
Mortality within 30 days after admission, by hour of admission. Relative change in hazard ratios with 95% confidence interval is displayed. Number of admissions per hour is displayed on the left. Adjusted for sex, age, day-of-the-week, day-after-holiday, month, and year.

Figures 3 and 4 show the average relative difference in hazard ratios of mortality within 30 days of admission, by day and hour of admission. There were only slight differences in mortality between admissions on weekdays. Compared with admissions on Mondays, admissions on Sundays and holidays had a slightly increased mortality. The hazard ratios were 1.1 (95% confidence interval [CI] 0.97–1.2) for Sundays, and 1.2 (CI 0.98–1.4) for holidays, compared with Monday admissions. Mortality after day of discharge was lowest for Monday discharges. There was about a 10% increased mortality for all weekday discharges as compared with Mondays. Compared with discharges on Mondays, discharges on Saturdays, Sundays, and holidays had a substantially increased mortality. The hazard ratio was 1.5 (CI 1.3–1.8) for Saturday discharges, 1.6 (CI 1.3–2.0) for Sunday discharges, and 1.4 (CI 1.1–1.8) for holiday discharges, compared with Monday discharges.

Time of day of admissions was largely unrelated to mortality, except for a very marked spike in the early morning. Admissions between 6:00 am and 8:00 am had a 40% increased risk, and admissions between 8:00 am and 11:00 am show a somewhat elevated risk.

**Figure 5.**
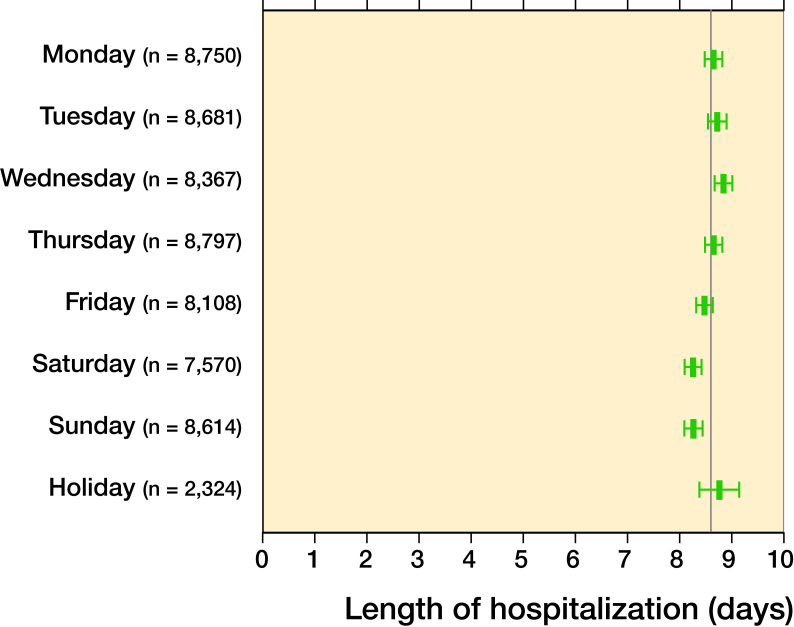
Estimated length of hospitalization according to day of admission, with 95% margins. Number of admissions per category in the data are displayed on the left. Adjusted for sex, age, admission hour, day-after-holiday, month, and year.

Figure 5 shows the estimated length of hospitalization by day of admission. Patients admitted on weekends had shorter stays, varying between 7.7 and 8.1 days. Patients admitted on weekdays except Fridays stayed longer (between 8.1 and 8.6 days), and those admitted on Fridays had a slightly shorter stay.

## Discussion

Our results suggest that there might be other timing effects in addition to the well-studied weekend effect. We find slightly increased mortality for patients admitted on Sundays and holidays, but not Saturdays, whereas early morning admissions and weekend discharges are associated with much higher mortality.

The finding of fewer hip fracture admissions at weekends is in line with a study from the UK (Johansen and Boulton 2017), which reports this as particularly true for in-hospital fractures. Because it is an acute condition, admissions for hip fractures are not likely to be postponed until weekdays if the fracture occurs during weekends. This is reflected in the fact that the number of admissions was not higher on Mondays compared with other weekdays.

There was increased mortality for patients admitted between 6:00 and 8:00 am. With this finding we have, to some extent, pinpointed a time of day when frail and severely ill patients are especially vulnerable. We did not have data on time of fracture or surgery, which would be a natural starting point for investigations into the causes of this finding. Our finding of increased mortality for early morning arrivals has not been reported before, since studies usually consider a dichotomous outcome of out-of-hours versus working-hours (Bray et al. 2016).

The finding of markedly higher mortality for patients discharged on weekends or holidays is in line with another recent study (Sayers et al. 2017), reporting 50% higher mortality for patients discharged on Sundays compared with other days of the week. While fewer patients are discharged during weekends and holidays, weekend discharges still account for about 20% of the discharge volume from an ordinary day in our material. The association could be confounded by discharges of frail patients to primary health services. However, we did not have any data on discharge destination to investigate this. Alternative explanations for the elevated mortality could be sought in reduced hospital and community staffing, which could impact the timeliness of discharge, accuracy of discharge instructions, and medication reconciliation (Cloyd et al. 2015). Discharging patients from hospital is a complex task, which requires clear communication among physicians, nurses, case managers, social workers, and the family (Hesselink et al. 2012).

The shorter length of stay for patients admitted on weekends, and to a certain extent Fridays, may be due to discharge routines, e.g., discharging patients before the weekend. An average stay of around 8 days implies that patients admitted on Fridays and weekends would, on average, be under consideration for discharge the following Friday.

We have not investigated changes over time in detail. However, it has been pointed out that despite a decline in the hip fracture rate over the past 15 years, the absolute number of fractures may increase as the number of older individuals grows (Søgaard et al. 2016). A recent Danish study (Pedersen et al. 2017) on incidence rates of hip fracture and mortality found that the decline in incidence was not seen in all patient groups.

Our findings suggest the importance of well-organized hospital treatment of hip fracture patients at any time of week and day. The results indicate the need for a wider scope on timing effects. The narrow focus on weekday versus weekend effects might have distracted attention from important factors related to time of day, but also discharge from hospital treatment.

### Supplementary data

Figure 1, and Tables 1–7 with descriptive statistics and results from regression analyses are available as supplementary data in the online version of this article, http://dx.doi.org/10.1080/17453674.2018.1533769

### Availability of data and material

The data of this study are available from the Norwegian patient registry, but restrictions apply to availability. These data were used under license for the current study, and are not publicly available.

AA, JHB, SMN, and MTM conceived the study. AA performed the data analysis and visualizations. AA, SMN, and MTM wrote the first draft of the manuscript. All authors interpreted the data and edited the manuscript. AA, SMN, and MTM are joint first authors.

*Acta* thanks Klaus Hindsø and Alma B Pedersen for help with peer review of this study.

## Supplementary Material

Supplemental Material
