# Supplementary material for: Time of admission and mortality after hip fracture: a detailed look at the weekend effect in a nationwide study of 55,211 hip fracture patients in Norway
Source: Acta Orthop. 2018 Nov 6;89(6):610–4. doi: 10.1080/17453674.2018.1533769 (PMC6319186; doi:10.1080/17453674.2018.1533769)
Supplement: Supplemental Material [file IORT_A_1533769_SM8240.pdf]

Supplementary data

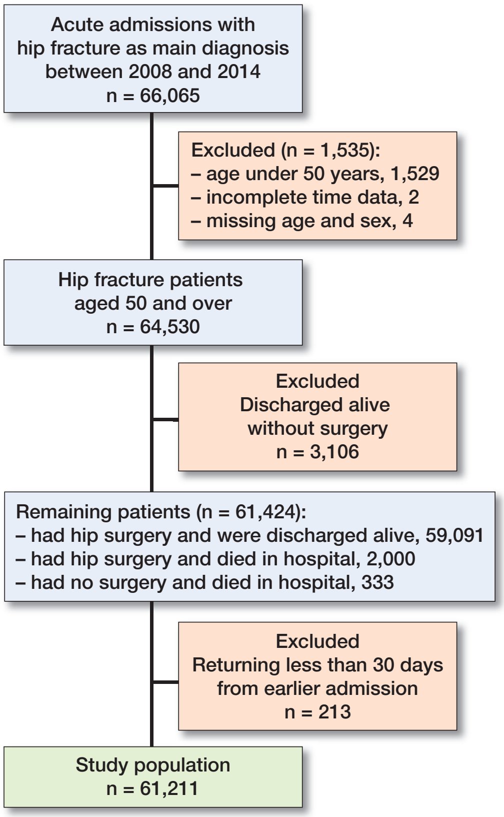

Figure 1. Flow chart of the study cohort.

Table 1. Results from regression analyses

	30-day mortality, on admissions Relative risk (95% CI)	30-day mortality, on discharge Relative risk (95% CI)	Length of hospital-ization (days) Relative change (95% CI)
Sunday	1.08 (0.97–1.20)	1.62 (1.33–1.96)	0.96 (0.93–0.98)
Monday	Ref.	Ref.	Ref.
Tuesday	0.94 (0.84–1.04)	1.14 (1.00–1.30)	1.01 (0.98–1.04)
Wednesday	0.94 (0.85–1.05)	1.12 (0.97–1.28)	1.02 (0.99–1.05)
Thursday	0.96 (0.86–1.06)	1.16 (1.01–1.33)	1.00 (0.97–1.03)
Friday	1.05 (0.95–1.16)	1.15 (1.01–1.31)	0.98 (0.95–1.01)
Saturday	0.94 (0.85–1.05)	1.52 (1.27–1.81)	0.95 (0.93–0.98)
Holiday	1.18 (0.98–1.41)	1.44 (1.13–1.84)	1.01 (0.97–1.06)
Hour 0	0.87 (0.71–1.07)	0.73 (0.38–1.40)	1.06 (1.01–1.11)
Hour 1	1.06 (0.86–1.31)		1.01 (0.96–1.07)
Hour 2	0.95 (0.74–1.20)		1.04 (0.98–1.10)
Hour 3	1.04 (0.81–1.32)		1.05 (0.98–1.13)
Hour 4	1.14 (0.88–1.47)	0.88 (0.45–1.70)	0.98 (0.91–1.06)
Hour 5	1.09 (0.83–1.43)		1.00 (0.94–1.08)
Hour 6	1.41 (1.10–1.80)		1.06 (0.98–1.15)
Hour 7	1.37 (1.07–1.75)		1.02 (0.94–1.11)
Hour 8	1.21 (0.97–1.51)	0.94 (0.48–1.84)	0.96 (0.90–1.01)
Hour 9	1.15 (0.96–1.37)		1.00 (0.95–1.06)
Hour 10	1.18 (1.02–1.38)		0.93 (0.90–0.97)
Hour 11	1.04 (0.90–1.21)		0.98 (0.94–1.02)
Hour 12	0.99 (0.85–1.15)	Ref.	0.96 (0.93–1.00)
Hour 13	0.99 (0.85–1.15)		1.01 (0.97–1.05)
Hour 14	Ref.		Ref.
Hour 15	0.94 (0.81–1.10)		1.02 (0.99–1.06)
Hour 16	0.99 (0.84–1.15)	0.43 (0.13–1.41)	1.01 (0.98–1.05)
Hour 17	0.94 (0.80–1.10)		0.99 (0.95–1.03)
Hour 18	0.89 (0.76–1.05)		1.01 (0.97–1.05)
Hour 19	0.91 (0.77–1.08)		1.03 (0.98–1.07)
Hour 20	1.03 (0.87–1.21)	0.67 (0.34–1.30)	1.02 (0.98–1.06)
Hour 21	1.03 (0.87–1.21)		1.02 (0.98–1.07)
Hour 22	0.97 (0.81–1.16)		1.08 (1.03–1.13)
Hour 23	0.88 (0.73–1.06)		1.04 (0.99–1.09)
January	Ref.	Ref.	Ref.
February	1.06 (0.93–1.21)	1.00 (0.83–1.19)	1.00 (0.96–1.03)
March	1.06 (0.93–1.21)	1.10 (0.93–1.32)	0.99 (0.95–1.02)
April	1.01 (0.88–1.16)	0.97 (0.80–1.17)	0.97 (0.94–1.00)
May	0.95 (0.82–1.09)	0.99 (0.82–1.19)	0.98 (0.95–1.01)
June	1.02 (0.89–1.17)	0.91 (0.75–1.11)	0.94 (0.91–0.97)
July	0.97 (0.85–1.12)	0.98 (0.82–1.19)	0.93 (0.90–0.96)
August	1.08 (0.94–1.23)	1.03 (0.86–1.24)	0.98 (0.94–1.01)
September	0.97 (0.85–1.12)	0.93 (0.76–1.12)	0.99 (0.96–1.03)
October	1.02 (0.89–1.16)	0.93 (0.77–1.12)	1.00 (0.97–1.04)
November	1.07 (0.94–1.22)	1.01 (0.83–1.21)	0.98 (0.95–1.02)
December	1.05 (0.92–1.19)	0.95 (0.79–1.14)	0.93 (0.90–0.96)
Year 2008	Ref.	Ref.	Ref.
Year 2009	1.00 (0.90–1.11)	1.04 (0.89–1.21)	0.95 (0.92–0.97)
Year 2010	0.95 (0.85–1.05)	0.96 (0.82–1.12)	0.90 (0.87–0.92)
Year 2011	0.94 (0.85–1.05)	0.96 (0.82–1.11)	0.83 (0.81–0.85)
Year 2012	0.96 (0.87–1.07)	0.89 (0.77–1.04)	0.65 (0.64–0.67)
Year 2013	0.91 (0.82–1.02)	0.85 (0.73–0.98)	0.62 (0.60–0.63)
Year 2014	1.00 (0.90–1.11)	0.93 (0.80–1.08)	0.61 (0.59–0.62)
Male	Ref.	Ref.	Ref.
Female	0.45 (0.43–0.48)	0.43 (0.40–0.47)	0.92 (0.90–0.93)
Age	1.08 (1.08–1.09)	1.09 (1.08–1.09)	1.00 (1.00–1.00)
Day after holiday	0.78 (0.65–0.94)	0.73 (0.58–0.93)	0.96 (0.92–1.00)

Table 2. Characteristics of study cohort

	Dead 30 days after admission Yes (n = 4,914)	No (n = 56,297)
Sex, n (%)		
Female	2,820 (57)	40,125 (71)
Male	2,094 (43)	16,176 (29)
Average age in years (SD)	87 (7)	81 (10)
Main diagnosis, n (%)		
Collum femoris fracture	2,891 (59)	34,175 (61)
Pertrochanteric fracture	1,749 (36)	18,856 (33)
Subtrochanteric fracture	274 (5)	3,266 (6)
Length of hospitalization (days) [a]		
Average	6.8	8.8
25% quantile	2.9	4.1
Median	4.9	6.6
75% quantile	8.7	10.6

[a] For patients discharged alive.

Table 3. Number of hip fractures, incidence rates, and case fatality 2008–2014 [a]

Year	Hip fractures	Deaths within 30 days from admission	Incidence rate per 1,000 persons per year	30 days case fatality (%)
2008	8,973	725	5.7	8.1
2009	8,754	717	5.4	8.2
2010	8,580	673	5.2	7.8
2011	8,827	702	5.3	8.0
2012	8,774	709	5.2	8.1
2013	8,823	671	5.1	7.6
2014	8,480	717	4.8	8.5
Total	61,211	4,914	5.2	8.0

[a] Data from Statistics Norway have been used to estimate population in the age group in order to estimate the incidence rate.

Table 4. Number of hip fractures, distribution, case fatality, age, sex, and main diagnosis per day of admission

Day of admission	Hip fractures	Fraction of total (%)	Deaths within 30 days from admission	30 days case fatality (%)	Female (%)	Average age (years)	% of fractures in categories S72.0, S27.1, S72.2
Monday	9,018	15	733	8.1	70	82	61, 33, 6
Tuesday	8,944	15	689	7.7	70	82	61, 34, 5
Wednesday	8,834	14	682	7.7	70	82	60, 34, 6
Thursday	8,853	14	699	7.9	71	82	60, 34, 6
Friday	9,098	15	765	8.4	70	82	61, 33, 6
Saturday	8,465	14	646	7.6	70	81	60, 34, 6
Sunday	7,999	13	700	8.8	70	81	60, 34, 6
Total	61,211	100	4,914	8.0	70	82	60, 34, 6

Table 5. Number of hip fractures, distribution, case fatality, age, sex, and main diagnosis per day of discharge

Day of discharge	Hip fractures	Fraction of total (%)	Deaths within 30 days from admission	30 days case fatality (%)	Female (%)	Average age (years)	% of fractures in categories S72.0, S27.1, S72.2
Monday	11,722	19	820	7.0	71	82	60, 34, 6
Tuesday	10,814	18	777	7.2	70	82	60, 34, 6
Wednesday	10,297	17	744	7.2	71	82	60, 34, 6
Thursday	10,144	17	781	7.7	70	82	60, 34, 6
Friday	11,665	19	845	7.2	70	81	61, 33, 6
Saturday	3,690	6	496	13.4	70	81	62, 33, 5
Sunday	2,879	5	451	15.7	67	82	62, 33, 5
Total	61,211	100	4,914	8.0	70	82	60, 34, 6

Table 6. Number of hip fractures, distribution, case fatality, age, sex, and main diagnosis per hour of admission

Hour of admission	Hip fractures	Fraction of total (%)	Deaths within 30 days from admission	30 days case fatality (%)	Female (%)	Average age (years)	% of fractures in categories S72.0, S27.1, S72.2
00:00 to 01:00	1,837	3	136	7.4	71	82	56, 37, 7
01:00 to 02:00	1,361	2	118	8.7	72	82	57, 36, 7
02:00 to 03:00	1,063	2	89	8.4	72	82	55, 38, 7
03:00 to 04:00	947	2	82	8.7	72	82	55, 38, 7
04:00 to 05:00	770	1	76	9.9	67	82	52, 41, 7
05:00 to 06:00	705	1	65	9.2	72	82	52, 42, 6
06:00 to 07:00	705	1	80	11.3	73	82	51, 41, 8
07:00 to 08:00	736	1	80	10.9	74	82	52, 40, 8
08:00 to 09:00	1,038	2	100	9.6	72	82	54, 39, 7
09:00 to 10:00	2,069	3	200	9.7	70	82	59, 36, 5
10:00 to 11:00	3,224	5	320	9.9	70	83	60, 34, 6
11:00 to 12:00	3,941	6	333	8.4	69	82	62, 33, 5
12:00 to 13:00	4,343	7	344	7.9	69	82	63, 32, 5
13:00 to 14:00	4,483	7	344	7.7	69	81	64, 31, 5
14:00 to 15:00	4,574	7	354	7.7	70	81	62, 32, 6
15:00 to 16:00	4,342	7	318	7.3	69	81	62, 32, 6
16:00 to 17:00	3,972	6	301	7.6	69	81	63, 31, 6
17:00 to 18:00	3,668	6	268	7.3	71	81	62, 33, 5
18:00 to 19:00	3,550	6	251	7.1	69	81	63, 32, 5
19:00 to 20:00	3,100	5	220	7.1	70	81	61, 33, 6
20:00 to 21:00	3,078	5	244	7.9	71	81	61, 33, 6
21:00 to 22:00	2,947	5	241	8.2	72	82	61, 34, 5
22:00 to 23:00	2,474	4	190	7.7	72	82	58, 36, 6
23:00 to 24:00	2,284	4	160	7.0	71	82	59, 35, 6
Total	61,211	100	4,914	8.0	70	82	60, 34, 6

Table 7. Number of hip fractures, distribution, case fatality, age, sex and main diagnosis per month of admission

Month of admission	Hip fractures	Fraction of total (%)	Deaths within 30 days from admission	30 days case fatality (%)	Female (%)	Average age (years)	% of fractures in categories S72.0, S27.1, S72.2
January	6,023	10	477	7.9	81	68	60, 34, 6
February	5,438	9	448	8.2	81	68	61, 33, 6
March	5,510	9	458	8.3	81	70	61, 33, 6
April	4,685	8	382	8.2	82	71	60, 34, 6
May	4,730	8	368	7.8	82	71	62, 32, 6
June	4,523	7	359	7.9	82	71	61, 33, 6
July	4,741	8	363	7.7	82	72	60, 33, 7
August	4,782	8	408	8.5	82	71	61, 34, 5
September	4,643	8	364	7.8	82	71	61, 33, 6
October	4,920	8	399	8.1	82	71	60, 34, 6
November	5,042	8	420	8.3	81	69	59, 35, 6
December	6,174	10	468	7.6	81	69	61, 34, 5
Total	61,211	100	4,914	8.0	82	70	60, 34, 6
